# Reproductive and endocrinological effects of Benign Prostatic Hyperplasia and finasteride therapy in dogs

**DOI:** 10.1038/s41598-020-71691-7

**Published:** 2020-09-09

**Authors:** Daniel S. R. Angrimani, Maíra M. Brito, Bruno R. Rui, Marcílio Nichi, Camila I. Vannucchi

**Affiliations:** Department of Animal Reproduction, School of Veterinary Medicine and Animal Science, University of São Paulo, Av. Prof. Orlando Marques de Paiva, 87, São Paulo, 05508-270 Brazil

**Keywords:** Infertility, Endocrine reproductive disorders

## Abstract

Benign prostatic hyperplasia (BPH) is one of the most important reproductive disorders in aging dogs. Therapeutic measures include orchiectomy and pharmacological treatment, leading to reduction of prostate volume and clinical signs. One of the most common drugs used in BPH treatment is finasteride, but data regarding its possible side effects are scarce. Thus, the aim of this study was to evaluate the effects of BPH and short-term (2 months) finasteride therapy on clinical, endocrinological, and reproductive parameters in dogs. Dogs were allocated into four experimental groups: Non-affected (n = 5), BPH (n = 5), Non-Affected-Finasteride (n = 5) and BPH-Finasteride (n = 5) groups. Dogs were evaluated monthly during 2 months by a complete breeding soundness examination, B-mode ultrasound and Doppler ultrasonography of the testicular artery, hormonal profile (testosterone, estrogen and dihydrotestosterone) and oxidative profile of the prostatic fluid. After 2 months, dogs were gonadectomized and testicles were subjected to histologic analysis. Finasteride treatment reduced dihydrotestosterone concentrations, without negative influence on semen quality and also reverted testicular hemodynamics changes of BPH. On the other hand, BPH was accompanied by significant changes in testosterone and estrogen concentrations and semen quality, mainly related to sperm kinetics alterations. In conclusion, BPH dogs have important hormonal and sperm alterations, however, short-term finasteride treatment (2 months) was able to reduce overall effects of BPH, thus representing a method of therapy for BPH treatment.

## Introduction

Benign prostatic hyperplasia (BPH) is considered the most common prostatic disease in dogs^1,2^. Although it affects both elderly men and uncastrated dogs above 5 years old^3^, BPH can also be diagnosed in young breeding males^4^. The disease can have a silent course, being asymptomatic in several cases, but is commonly related to tenesmus, dysuria, frequent urination, hematuria and hematospermia^5,6^.

The etiology of BPH is still not completely elucidated. It is known that the main trigger factor is an imbalance between testosterone and estrogen synthesis due to senescence (lower concentrations of androgen and higher concentrations of estrogen)^7,8^. The lack of endocrine equilibrium leads to a quantitative increase in prostate androgen receptors, upregulating testosterone action^5,9^, which is the pivotal reason for a markedly conversion of testosterone into dihydrotestosterone (DHT) by the enzymatic action of 5α-reductase^7,9^. Increased DHT concentrations provoke several prostatic alterations, such as hypertrophy and hyperplasia of glandular cells, angiogenesis^9^ and even local oxidative stress in men^10^ and dogs^11^.

Reproductive hormonal imbalance and prostate morpho-functional changes are both referred to alter sperm quality of BPH dogs^8,12,13^, standing for altered sperm kinetics, high percentage of morphologically abnormal spermatozoa and lower sperm DNA integrity^14,15^. Decreased semen quality may impact fertility and also negatively influence semen biotechnology^16^. Thus, increasing our understanding on the reproductive effects of BPH in dogs can allow a more precise and effective clinical approach.

Azasteroid finasteride is the best described drug therapy for BPH in men^17^. Finasteride is a synthetic inhibitor of 5α-reductase and acts by blocking its enzymatic action, therefore reducing DHT levels and ultimately, decreasing prostatic size and clinical signs related to BPH^18–20^. On the other hand, finasteride therapy in men has been demonstrated to cause erectile dysfunction, loss of libido and poor sperm quality, such as oligospermia and azoospermia^21–23^. In addition, Vidigal et al.^24^ showed reduction in spermatogenesis rate and seminiferous tubules atrophy in hamsters, indicating a possible irreversible and negative effect of finasteride therapy. In dogs, decreased sperm quality was only observed after long term treatment with finasteride^4^. On the other hand, Iguer-Ouada; Verstegen^25^, observed increased sperm concentration whilst prostate reduction with finasteride treatment. Hence, the effects of both BPH and subsequent finasteride treatment on sperm output and quality should be analyzed by clinical trials. The obtained knowledge may allow for the safe use of stud dogs in reproductive programs while these dogs are under the finasteride therapy for BPH.

In this context, the purpose of this study was to evaluate the influence of BPH and/or finasteride therapy on sperm features, testicular morpho-function and hormonal profile in dogs.

## Results

No statistical interaction among BPH, finasteride treatment and time-points (0, 30 and 60 days) was observed.

After 30 days of finasteride treatment, all BPH-Finasteride dogs fully recovered from the initial clinical signs (tenesmus, hematospermia, hematuria or dysuria). Dogs of the BPH Group remained clinically stable, without any further complications.

**Figure 1:**
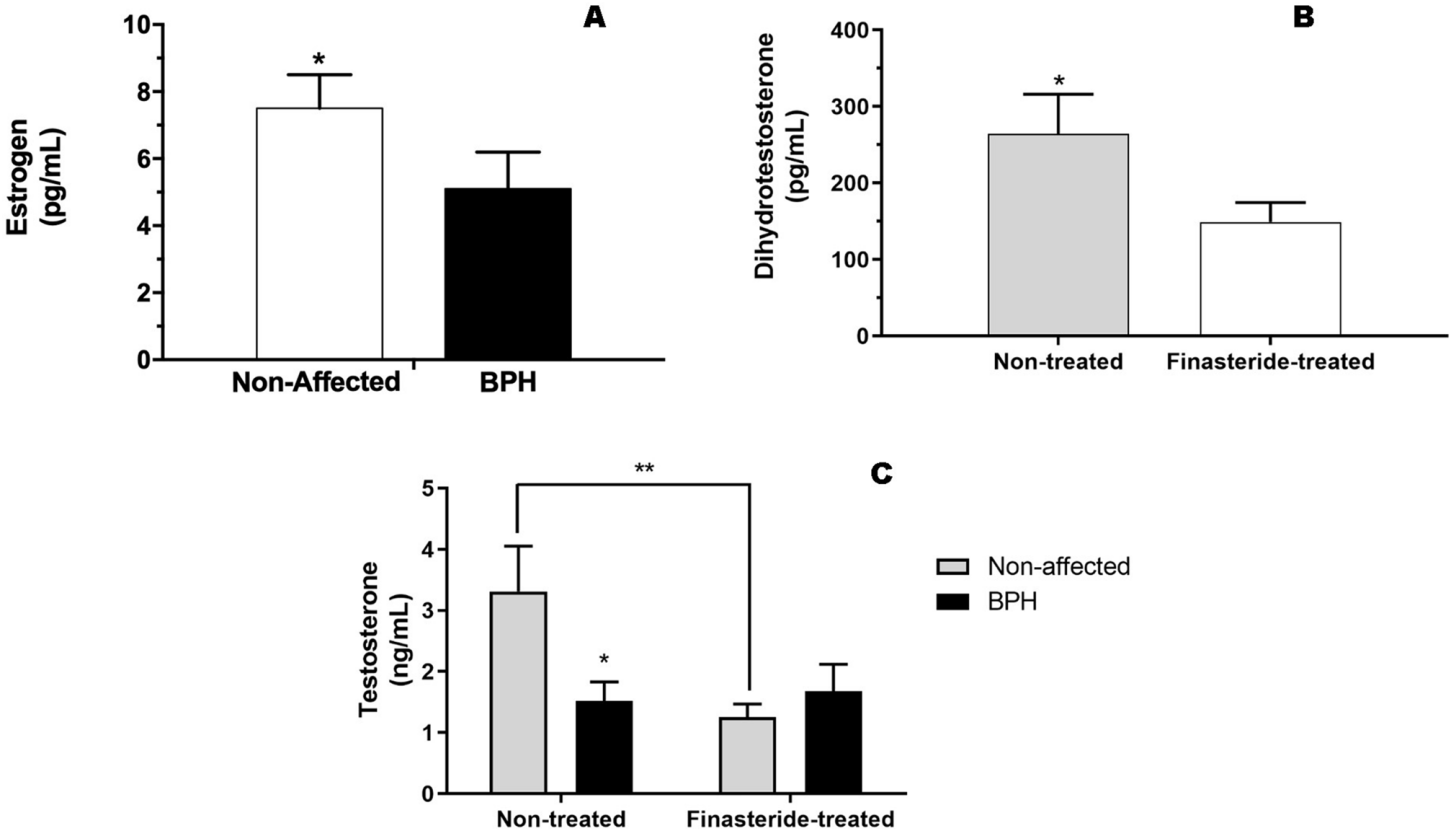
**(A)** Estrogen (pg/mL) concentration in Non-affected and BPH dogs. (**B)** Dihydrotestosterone (pg/mL) concentration in Non-treated and Finasteride-treated dogs. (**C)** Testosterone concentration (ng/mL) of Non-affected, BPH, Non-treated and Finasteride-treated dogs. *Indicate significant difference (P < 0.05). **Indicate significant difference between Non-affected and Non-affected-Finasteride treated dogs (P < 0.05).

A significant interaction between BPH and finasteride treatment was observed for testosterone assay (P < 0.04). Non-affected dogs had higher (P < 0.05) testosterone concentrations (3.3 ± 0.7 ng/mL) compared to BPH dogs (1.5 ± 0.3 ng/mL—Fig. 1C). Moreover, non-affected dogs had higher (P < 0.05) testosterone concentrations than non-affected-finasteride dogs (1.2 ± 0.2 ng/mL—Fig. 1C). Furthermore, we observed a positive correlation between testosterone concentration and total sperm motility (r = 0.44, P = 0.01) and progressive sperm motility (r = 0.39, P = 0.02) in the BPH Group. For the estrogen assay, non-affected dogs had higher (P < 0.05) estrogen concentrations (7.5 ± 1 pg/mL) compared to BPH dogs (5.1 ± 1.1 pg/mL), regardless of finasteride treatment or moment of evaluation (Fig. 1A). Finasteride-treated dogs had lower (P < 0.05) dihydrotestosterone concentrations (149.3 ± 25.1 pg/mL) in comparison to non-treated dogs (264.5 ± 51.6 pg/mL), regardless of BPH occurrence (Fig. 1B).

**Figure 2:**
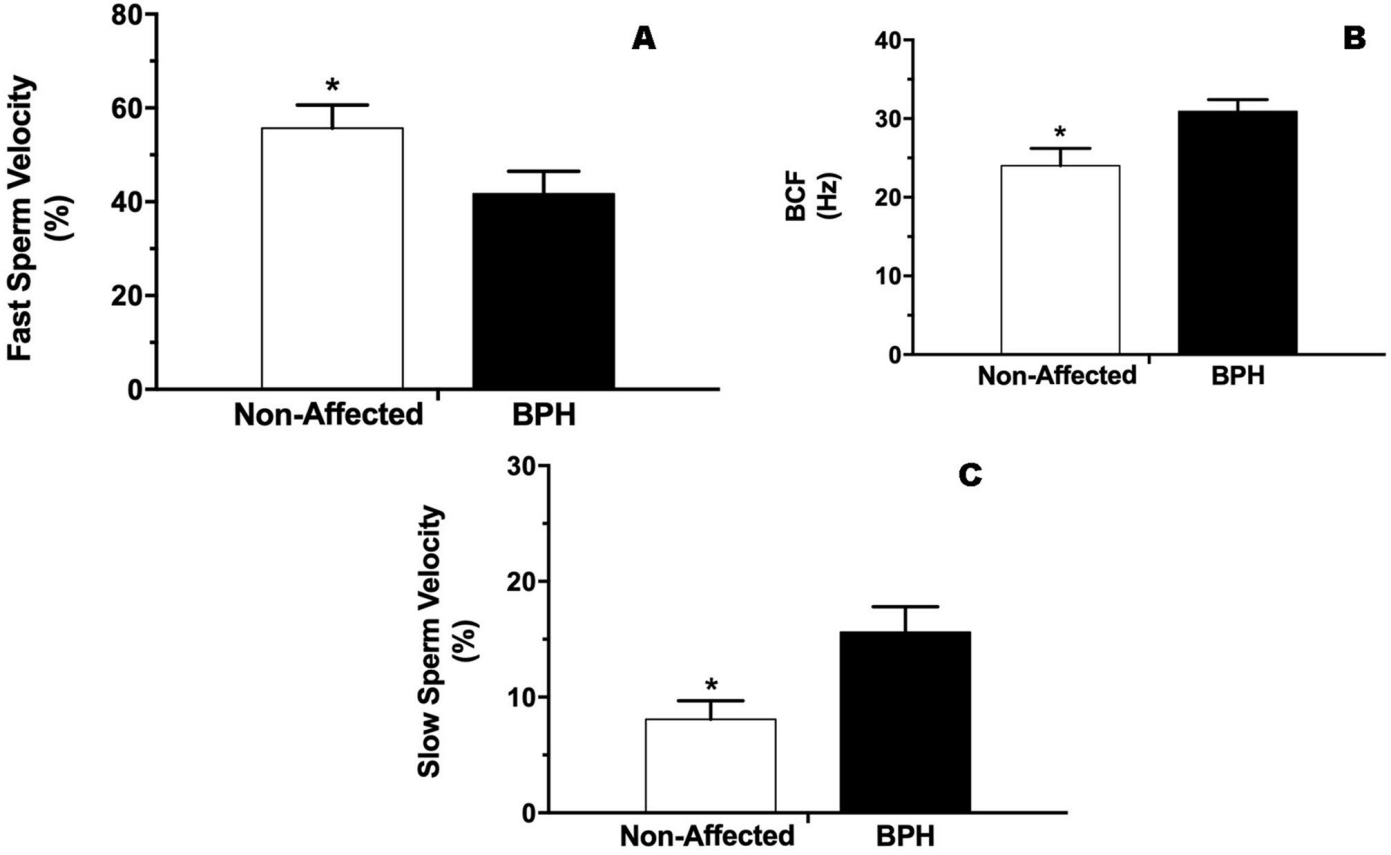
**(A)** Fast sperm velocity (%), **(B)** sperm beat cross-frequency (Hz) and **(C)** slow sperm velocity (%) in Non-affected and BPH dogs. *Indicate significant difference (P < 0.05).

Percentage of sperm with fast velocity was higher (P < 0.05) in non-affected dogs (47.6 ± 4.3%) than BPH dogs (41.7 ± 4.8%), regardless of finasteride treatment (Fig. 2A). Conversely, sperm beat cross-frequency (BCF) was lower (P < 0.05) in non-affected dogs (24 ± 2.2%) compared to BPH dogs (30.9 ± 1.5%), regardless of finasteride treatment (Fig. 2B). Furthermore, percentage of slow sperm was lower (P < 0.05) in non-affected dogs (8.1 ± 1.6%) in comparison to BPH dogs (15.6 ± 2.2%) (Fig. 2C). Concerning the sperm kinetic analysis, no differences were verified throughout finasteride treatment (Table 1).

**Table 1:** Mean and standard error (X ± SE) of computer analysis of motility (CASA) in the Non-treated and Finasteride-treated groups.

	Non-treated	Finasteride-treated	P
Total motility (%)	66 ± 4.1	65 ± 5.4	0.8
Progressive motility (%)	40.6 ± 4.1	42.8 ± 4.5	0.7
Percentage of fast sperm velocity (%)	46.6 ± 4.7	50.5 ± 5.3	0.5
Percentage of medium sperm velocity (%)	19.5 ± 2.2	14.3 ± 1.9	0.08
Percentage of slow sperm velocity (%)	13.6 ± 2	10.3 ± 2	0.2
Percentage of static sperm (%)	20.3 ± 2.9	24.6 ± 5.3	0.5
Sperm velocity average pathway (μm/s)	95.7 ± 5.9	98.8 ± 6.4	0.7
Sperm velocity straight line (μm/s)	84.7 ± 5.8	87.2 ± 5.9	0.7
Sperm curvilinear velocity (μm/s)	134.5 ± 7.5	141.3 ± 8.9	0.5
Sperm beat cross-frequency (Hz)	27.9 ± 1.6	27.3 ± 2.3	0.8
Amplitude of lateral head displacement (μm/s)	5.4 ± 0.2	5.7 ± 0.3	0.5
Sperm straightness (%)	85.2 ± 1.5	83.1 ± 3.2	0.5
Sperm linearity (%)	63.7 ± 2.3	61.2 ± 3.1	0.6

**Figure 3:**
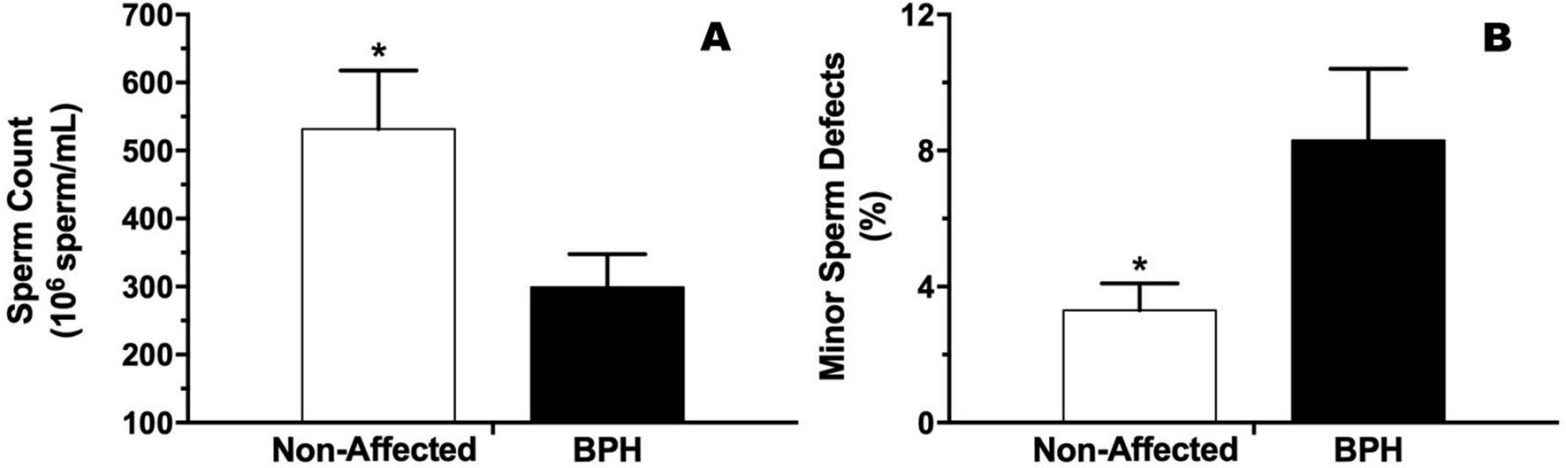
**(A)** Sperm count (sperm/mL) and **(B)** minor sperm defects (%) in Non-affected and BPH dogs. *Indicate significant difference (P < 0.05).

BPH dogs had lower (P < 0.05) sperm count (299.1 ± 48.8 sperm/mL) than non-affected dogs (531.6 ± 86 sperm/mL—Fig. 3A). Conversely, BPH dogs had higher percentage of minor sperm defects (8.3 ± 2.1%) compared to non-affected dogs (3.3 ± 0.6%—Fig. 3B).

**Figure 4:**
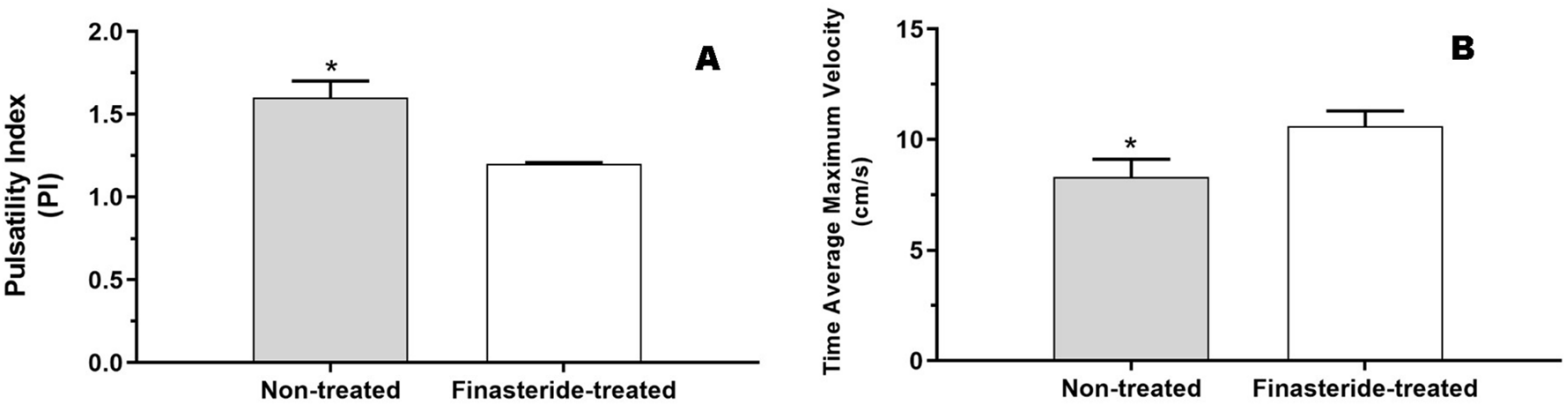
**(A)** Pulsatility index (PI) and **(B)** time average maximum velocity (TAMAX) in Non-treated and Finasteride-treated dogs. *Indicate significant difference (P < 0.05).

Regarding hemodynamic analysis of the testicular artery, finasteride-treated dogs (1.2 ± 0.008) had lower (P < 0.05) pulsatility index compared to non-treated animals (1.6 ± 0.1), regardless of BPH occurrence (Fig. 4A). Conversely, time average maximum velocity (TAMAX) was higher (P < 0.05) in finasteride-treated dogs (10.6 ± 0.7 cm/s) in comparison to non-treated groups (8.3 ± 0.8 cm/s) (Fig. 4B).

Regardless of the experimental group, all testicular samples (right and left testicles) presented mild to moderate cellularity of the seminiferous tubules, with scarce areas of focal high cellularity. Seminiferous tubules were filled with acellular eosinophilic amorphous material in the absence of inflammatory infiltrate or blood. All the evaluated groups were classified as score 0, that means, no lesions were observed.

No differences between groups were verified for the oxidative status of the prostatic fluid (Table 2). No time-point differences (Day 0, Day 30 and Day 60; P > 0.05) were observed throughout the experiment.

**Table 2:** Effect of Benign Prostatic Hyperplasia (Non-affected vs. BPH) and finasteride treatment (Non-treated vs. Finasteride-treated) on the analysis of oxidative profile of prostatic fluid.

	Non-affected	BPH	P	Non-treated	Finasteride-treated	P
TBARS (ng/TBARS)	469.8 ± 63.6	377.8 ± 53.4	0.2	386.9 ± 56.9	467 ± 61.5	0.2
Superoxide dismutase (SOD-IU ml^-1^)	30.7 ± 4.6	23.3 ± 3.8	0.4	28.5 ± 5.1	25.3 ± 3.2	0.7
Glutathione peroxidase (GPx-IU ml^-1^)	247.1 ± 38.4	196.7 ± 32.8	0.3	199.5 ± 33.4	244.2 ± 38	0.6

## Discussion

In the present study, we evaluated the influence of benign prostatic hyperplasia and/or treatment with finasteride in dogs, by means of a complete breeding soundness examination. Benign prostatic hyperplasia is a homologous condition in men and dogs, and the most commonly diagnosed prostatic disorder in aged and intact dogs^26,27^. Although castration is the treatment of choice, drug treatment with finasteride, a synthetic inhibitor of enzyme 5α-reductase, is one of the feasible medical alternatives for breeding dogs^17,28^.

Our data show that dogs with BPH have lower testosterone and estrogen concentration compared to unaffected dogs, as well as positive correlation between testosterone concentration and sperm kinects (total and progressive sperm motility). In fact, testosterone is the most important male hormone, acting on spermatogenesis, spermiogenesis and epididymal maturation^29^. Also, an appropriate hormonal environment is necessary for adequate spermatogenesis, including estrogen synthesis by the action of aromatase on Sertoli cells^30^. Thus, decreased concentration of testosterone and estrogen in dogs with BPH may directly affect ejaculate quality^13,29^. Thus, we assume that spermatogenesis dysfunction in BPH dogs is an indirect consequence of the hormone imbalance rather than a consequence of testicular morphological alterations, since no remarkable histological findings were observed that can justify the significant reduction in sperm concentration and higher percentage of minor sperm defects. Additionally, alterations of sperm kinetics in BPH dogs can be related to the decreased secretory function of Leydig cells.

Interestingly, high percentage of sperm beat cross-frequency (BCF) was observed in dogs with BPH, suggesting a kinetic pattern similar to premature sperm hyperactivation^31^. The excessive production of reactive oxygen species (ROS), changes in pH and osmolarity of the seminal fluid are common causes of premature hyperactivation^32^. However, oxidative stress and ROS generation are considered to occur only in severe and chronic prostatic disorders, with loss of prostatic parenchyma integrity^11,15^. In fact, we could not find significant differences in oxidative stress (TBARS) and antioxidant activity (SOD and GPx) within experimental groups. Thus, sperm kinetical alterations (i.e. BCF) are possibly related to altered biochemical characteristics of the prostatic fluid in dogs with BPH, such as ionic and binding protein conten^14,33,34^. Furthermore, we can also attribute the high percentage of sperm defects in BPH dogs to biochemical changes of the prostatic fluid, i.e., post-ejaculatory sperm alterations^13,14^. It is therefore possible to infer that biochemical changes of the prostatic fluid in BPH promote structural damage of post-ejaculated sperm cells.

Interestingly, BPH dogs had lower estrogen and testosterone concentrations compared to the Non-Affected Group, despite being within the normal range for adult dogs^35^. However, low testosterone concentrations are responsible for the neoformation of androgenic receptors in the prostate^36^, thus increasing testosterone action. On the other hand, non-affected dogs treated with finasteride had lower testosterone and DHT concentrations compared to the Non-treated Group. Thus, we assume that 5α-reductase blockage led to inhibition of testosterone conversion into DHT, ultimately resulting in negative feedback of Leydig cells by accumulative concentrations of testosterone^37^.

It is interesting to point out that finasteride treatment caused reduction of testicular artery blood volume (pulsatility index) and increase in blood flow velocity (time average maximum velocity), compared to untreated dogs. Therefore, DHT seems to act as a regulator of testicular vascular homeostasis, since low rates of testicular artery pulsatility index (PI) are related to efficiency in spermatogenesis^1^. In fact, according to Bergh et al.^38^ even a slight reduction in blood flow of the testicular artery may affect the initial process of spermatogenesis. Thus, we can suggest that the analysis of testicular artery PI can be used as a complementary marker of effective therapeutic effect during the course of finasteride treatment in BPH dogs.

Even though Vidigal et al.^24^ observed changes in testicular parenchyma of finasteride treated hamsters, our results could not prove such assertive in short-term treated dogs. However, we believe that 2 month course of finasteride therapy was not a sufficient period to promote significant testicular and seminal changes, similarly to the results of Iguer-Ouada; Verstegen^25^. Thus, in this respect, semen use from finasteride treated dogs is feasible for reproductive purposes and can be recommended during the first 2 months of therapy, which is a period enough for the assuagement of clinical signs and reduction in prostate size^19^.

In conclusion, dogs with benign prostatic hyperplasia present a hormonal imbalance, leading to seminal changes related mostly to sperm kinetics. On the other hand, the 2 months duration of finasteride therapy decreases hormonal profile of dihydrotestosterone and testosterone, resulting in vascular alteration of the testicular artery without promoting additional significant changes in sperm quality.

## Materials and methods

### Animals and experimental study

The current study was approved by the Bioethics Committee of the School of Veterinary Medicine and Animal Science—University of São Paulo (protocol number 7122171213). All experiments were performed in accordance with relevant guidelines and regulations.

Twenty dogs of several breeds, with body weights ranging from 10.2 to 29.5 kg and aged 5 to 13 years were selected and equally divided into four experimental groups according to BPH diagnosis and finasteride treatment. Presumptive diagnosis of BPH was based on clinical signs, prostatic secretion assessment and prostatic biometry by B-mode ultrasound^1,5^. The most common clinical signs of BPH dogs were hematospermia, hematuria, pollakiuria, dysuria and tenesmus, besides marked prostatomegaly. In addition, macroscopic examination of the prostatic fluid revealed reddish color and blood cells content. After examination, dogs were then randomly assigned to four experimental groups, composing a 2 × 2 factorial experimental design, considering BPH and finasteride therapy as the main experimental factors:

- Non-affected group (n = 5, Labrador Retriever, 2 Mixed Breed, Lhasa Apso, Labrador Retriever): dogs without prostatomegaly or any other clinical signs of BPH;
- BPH group (n = 5, Cocker Spaniel, Akita, Daschshund, Malinois Shepherd, Mixed Breed): dogs with a presumptive diagnosis of BPH (prostatomegaly and at least one clinical sign of BPH);
- BPH-Finasteride group (n = 5, Cocker Spaniel, German Shepherd, 2 Pit Bulls, Mixed Breed): dogs with a presumptive diagnosis of BPH subjected to oral finasteride therapy (5 mg/per animal/day of finasteride—Finasterida®/Medley®) during 2 months^39^;
- Non-affected-Finasteride group (n = 5, Akita, Labrador Retriever, Chow-Chow, Mixed Breed, English Bulldog): dogs without prostatomegaly or any other clinical signs of BPH subjected to oral finasteride therapy (5 mg/per animal/day of finasteride—Finasterida®/Medley®) during 2 months^39^;

To assure the appropriate sample size, an analysis was conducted with the SAS Power and Sample Size 12 (SAS Institute Inc., Cary, NC, EUA). A retrospective analysis of the data indicated there was a power of 0.99, which is considered an acceptable statistical power (at least 0.8). Hence, a minimum of 5 dogs per group were sufficient to demonstrate significant differences in the data. To ensure that the age of the dogs was not a determinant factor for the seminal analysis, dogs were equally distributed among groups. The mean age (± SEM) in each experimental group was as follow: 7 ± 1.2 years for the Non-affected group, 10 ± 1.9 years for the BPH group, 9 ± 2.1 years for the BPH-Finasteride group and 7 ± 1.4 years for the Non-affected-Finasteride group, with no significant statistical difference among groups.

All dogs were evaluated monthly during 2 months (Day 0, Day 30 and Day 60) by a complete breeding soundness examination, beginning at the first day of finasteride therapy or at the day of presumptive diagnosis of BPH.

After 60 days, bilateral orchiectomy was performed and testicles were subjected to histological analysis.

### Seminal evaluation

On days 0, 30 and 60, a semen sample was collected from each dog by digital manipulation of the penis. We collected only a single semen sample from each dog before ultrasonographic analysis, due to a remarkable influence of ejaculation on prostate vascularization as attested by Alonge et al.^40^. After the semen collection, the sperm-rich fraction was immediately analyzed for the following methods:

Volume of the sperm-rich fraction was measured using a graded test tube (mL). Sperm count (number of spermatozoa per mL) was analyzed through a haemocytometer counting chamber. For the assessment of sperm morphology, the eosin/nigrosin stain was performed. Briefly, 5 μL of semen and 5 μL of the previously prepared stain were placed in a glass slide and smeared. Sperm analysis was performed under light microscopy (Nikon, Eclipse E200, Japan) at × 1,000 magnification by counting 200 cells. Sperm morphological abnormalities were classified as major, minor and total, according to Barth and Oko^41^. In order to rule out a possible concurrent prostatitis, sperm smears were also analyzed for the presence of bacteria or leukocyte.

Sperm motility was evaluated using Computer Assisted Sperm Analysis (CASA, Hamilton-Thorne Ivos 12.3), with the following software settings: 38 °C of temperature, 10 fields acquired, 60 Hz frame rate, 75 minimum contrast, 6 pixels minimum cell size, 75% straightness (STR) threshold, 9.9 μm/s vap cut-off, 100 μm/s Prog. min VAP and 20 μm/s VSL cut-off. Fields of view were randomly selected and evaluated according to different patterns of motility^42^: velocity average pathway (VAP; μm/s), curvilinear velocity—VCL (μm/s), velocity straight line (VSL—μm/s), amplitude of lateral head displacement (ALH—μM), beat cross-frequency (BCF—Hz), straightness (STR—%), linearity (LIN—%), percentage of motility and progressive spermatozoa and percentage of rapid, medium, slow and static spermatozoa.

In order to analyze the integrity of sperm plasma and acrosome membrane, semen were analyzed by flow cytometry using the BD FACSCalibur equipment (Becton Dickinson, East Rutherford, NJ, USA). Sperm cells were previously stained with fluorescent probes according to the specific analysis, using a fixed sperm concentration of 188,000 cells diluted in 37.5 μL of TALP (Tyrode`s Albumin Lactate and Pyruvate). For the analysis of plasma and acrossomal membrane integrity, propidium iodide fluorescent probe (PI; 6 μM in TALP) was combined with *Psyllium agglutinin* conjugated with fluorescence isothiocyanate (FITC-PSA; 100 μg/mL of 1% sodium azide). The mixed probe solution was added to diluted sperm and incubated at 37 °C for 5 min. Subsequently, 300 μL of TALP was added and analyzed by flow cytometry, according to the protocol previously described^43,44^. For the analysis of sperm mitochondrial potential, 1 μL of 50 μg/mL JC-1 probe (5,5′, 6,6′ tetrachloro-1,1,3,3′-tetraethylbenzimidazolylcarbocyanine iodide) was added to diluted sperm. Samples were incubated at 37 °C for 10 min and then 300 μL of TALP were added and analyzed by flow cytometry, according to protocol previously described^45^.

### Oxidative profile of the prostatic fluid

Measurement of thiobarbituric acid reactive substances (TBARS) was performed to indirectly analyze lipid peroxidation, as previously developed by Ohkawa et al.^46^. To precipitate proteins, 300 μL of prostatic fluid and 600 μL of 10% trichloroacetic acid (TCA, Sigma Aldrich®) were mixed and centrifuged (20,817×*g* for 15 min at 5 °C). After centrifugation, 800 μL of the supernatant and 800 μL of 1% thiobarbituric acid (TBA, Sigma Aldrich®) in 0.05 N sodium hydroxide were placed into glass tubes, boiled in water bath (100 °C) for 10 min, and subsequently cooled in ice bath (0 °C) to stop the chemical reaction. The TBARS were then quantified using a spectrophotometer (Ultrospec 3300 pro®) at a wavelength of 532 nm. The results were compared to a standard curve previously prepared with a standard solution of malondialdehyde (MDA). The lipid-peroxidation index was described as nanograms of TBARS/mL.

Prostatic fluid was also subjected to the evaluation of antioxidant enzymes glutathione peroxidase (GPx) and superoxide dismutase (SOD) activity. Superoxide dismutase activity was assessed by indirect measurement of cytochrome c reduction by superoxide anion (O_2_^-^) through spectrometry at 550 nm wavelength at 25 °C for 5 min^30,31^. Glutathione peroxidase activity was measured by NADPH consumption, through the reaction between H_2_O_2_ and reduced glutathione (GSH), which is catalyzed by GPx and measured through spectrometry at 340 nm wavelength at 37 °C for 100 min^47,48^.

### Testicular ultrasonographic examination

Ultrasound evaluation of the testicles (right and left) was performed using a Mindray® M5 ultrasound (Shenzhen, China) equipped with a 7.5 MHz linear transducer. Dogs were placed in dorsal recumbency and trans-scrotal evaluation was performed without sedation. Parenchyma echogenicity was analyzed by conventional B-mode and testicular biometry was performed, measuring testicular length (L) and width (W) of the right and left testicles. Testis volume (cm^3^) was calculated considering the equation: L × W^2^ × 0.71^49^.

Testicular blood perfusion and velocity of blood flow were analyzed by Doppler ultrasonography. Testicular arteries were scanned at the spermatic funiculum region^50^ and pulsed-wave Doppler was used to characterize the waveform. Blood flow velocity parameters (peak systolic velocity—PSV—cm/s, end diastolic velocity—EDV—cm/s, time average maximum velocity—TAMAX—cm/s) and hemodynamic indices (Resistance Index—RI, Pulsatility Index—PI, Systolic:Diastolic velocity—S/D) were automatically calculated by the equipment software using mathematical formulas or Pourcelot index. Blood sample volume was positioned in the center of the vessel and the insonation angle was adjusted (less than 60°). A total of 9 stable waves of each testicular artery were obtained to calculate the average of each variable.

### Hormonal assay

Always during the morning period, blood samples were collected from the dogs by venous puncture into vacuum tubes without anticoagulant. The collection room was always maintained at 20 °C. Tubes were immediately centrifuged at 1,500×*g* for 10 min and serum was drawn-off and stored in microtubes at − 20 °C until analysis.

Hormonal assays were performed with the use of commercial radioimmunoassay kits for quantitative measurement of testosterone and estrogen (Beckman Coulter®), previously validated for dogs^51,52^, and competitive ELISA kits for dihydrotestosterone assay^53^, previously validated for dog. For estradiol, the intra-assay coefficients were 7.15% (high intra-assay) and 3.45% (low intra-assay) and the limit detection was 1.12 pg/mL. For testosterone, the intra-assay coefficient was 4.52% and the limit detection was 0.01 ng/mL. For dihydrotestosterone, the limit detection was 6.58 pg/mL and the coefficient of variation ranged from 3.03% (low intra-assay) and 9.86% (high intra-assay). All reactions were performed in duplicate.

### Testicular histologic analysis

After orchiectomy, 0.5 cm fragments of testicular parenchyma were washed in 0.9% physiological solution and immediately fixed in 10% buffered formaldehyde solution for 24 h, as previously described^49,54^. Then, fragments were stored in 70% alcohol, and then embedded in paraffin. Histological sections of 0.5 μm were submitted to dewaxing following the standard protocol. Hematoxylin–Eosin stain (HE) was used to evaluate testicular histomorphological aspect^49^. A minimum of 2 microscopic slides were analyzed from each testis. Analysis of the testicular parenchyma was based on an arbitrary scale of 0 to 3 (0: no lesions; 1: superficial and focal changes; 2: general heterogeneous aspect of the parenchyma and tissue fibrosis; 3: loss of testicular morphology with reduction of testis size).

### Statistical analysis

All data were evaluated using the SAS System for Windows (SAS Institute Inc., Cary, NC, USA). Effects of BPH, finasteride therapy and time-points (days 0, 30 and 60) and interactions between those factors were estimated by the repeated measures analysis of variance (Mixed Procedure of SAS). If no triple interactions (BPH X Finasteride X Time-points) occurred, the following interactions were considered: Time-points X Finasteride, Time-points X BPH and Finasteride X BPH. If no significant interactions were observed, the effect of groups was analyzed merging all time-points and conversely, time-points were compared combining all groups taking into account the Bonferroni correction (Proc Glimmix). Otherwise, comparisons were performed taking into account both effects. Differences between BPH and finasteride therapy were analyzed using parametric and non-parametric tests, according to the residual normality (Gaussian distribution) and variance homogeneity. Whenever one of these assumptions was not respected data were transformed. When no transformations were successful, non-parametric tests were employed. Thus, differences between BPH and Finasteride therapy were analyzed using Student t test (parametric variables) and Wilcoxon test (nonparametric variables). Results were described as untransformed means ± SE. The significance level used was P < 0.05. Moreover, Spearman correlation was used to calculate the relationship between variables.
